# Evaluating Binary Classifiers for Cardiovascular Disease Prediction: Enhancing Early Diagnostic Capabilities

**DOI:** 10.3390/jcdd11120396

**Published:** 2024-12-09

**Authors:** Paul Iacobescu, Virginia Marina, Catalin Anghel, Aurelian-Dumitrache Anghele

**Affiliations:** 1 Department of Computer Science and Information Technology, “Dunărea de Jos” University of Galati, 800201 Galati, Romania; paul.iacobescu@ugal.ro (P.I.); catalin.anghel@ugal.ro (C.A.); 2 Medical Department of Occupational Health, Faculty of Medicine and Pharmacy, “Dunărea de Jos” University of Galati, 800201 Galati, Romania; 3 Doctoral School of “Dunărea de Jos” University of Galati,800201 Galati, Romania; anghele_aurelian@yahoo.com

**Keywords:** cardiovascular diseases, artificial intelligence, machine learning, artificial intelligence in medical diagnosis

## Abstract

Cardiovascular disease (CVD) is a significant global health concern and the leading cause of death in many countries. Early detection and diagnosis of CVD can significantly reduce the risk of complications and mortality. Machine learning methods, particularly classification algorithms, have demonstrated their potential to accurately predict the risk of cardiovascular disease (CVD) by analyzing patient data. This study evaluates seven binary classification algorithms, including Random Forests, Logistic Regression, Naive Bayes, K-Nearest Neighbors (kNN), Support Vector Machines, Gradient Boosting, and Artificial Neural Networks, to understand their effectiveness in predicting CVD. Advanced preprocessing techniques, such as SMOTE–ENN for addressing class imbalance and hyperparameter optimization through Grid Search Cross-Validation, were applied to enhance the reliability and performance of these models. Standard evaluation metrics, including accuracy, precision, recall, F1-score, and Area Under the Receiver Operating Characteristic Curve (ROC-AUC), were used to assess predictive capabilities. The results show that kNN achieved the highest accuracy (99%) and AUC (0.99), surpassing traditional models like Logistic Regression and Gradient Boosting. The study examines the challenges encountered when working with datasets related to cardiovascular diseases, such as class imbalance and feature selection. It demonstrates how addressing these issues enhances the reliability and applicability of predictive models. These findings emphasize the potential of kNN as a reliable tool for early CVD prediction, offering significant improvements over previous studies. This research highlights the value of advanced machine learning techniques in healthcare, addressing key challenges and laying a foundation for future studies aimed at improving predictive models for CVD prevention.

## 1. Introduction

Cardiovascular diseases (CVDs) are the leading cause of global mortality, responsible for approximately 17.9 million deaths annually [1]. This broad category includes disorders of the heart and blood vessels, such as coronary heart disease and strokes. Heart attacks are a significant contributor to CVD-related deaths, particularly among individuals under 70 years old [1].

Behavioral risk factors, such as poor diet, physical inactivity, smoking, and excessive alcohol consumption, play an important role in the development of CVDs. These behaviors often lead to elevated blood pressure, blood glucose, and lipid levels, which are measurable “intermediate risk factors”, indicating an increased susceptibility to cardiovascular complications in primary care settings [1].

In addition to behavioral factors, genetic predispositions, environmental conditions, and lifestyle choices contribute to the development of CVDs. Among these, controllable risk factors such as hypertension, hyperlipidemia, physical inactivity, smoking, obesity, and high cholesterol levels are key contributors [2].

The diagnosis of CVD often involves a comprehensive medical history review, physical examination, and diagnostic tests such as electrocardiography, echocardiography, or myocardial perfusion imaging (MPI) [3]. Treatment options range from lifestyle interventions, such as proper nutrition and regular physical activity, to invasive surgical procedures aimed at mitigating disease progression.

The high prevalence of cardiovascular disease and its associated mortality highlights the critical importance of early detection and timely intervention to improve patient outcomes. Non-invasive imaging techniques, such as computed tomography (CT) and magnetic resonance imaging (MRI), offer detailed visualization of the heart and blood vessels, enabling clinicians to detect structural and functional abnormalities indicative of CVD [3]. These advancements support early diagnosis and effective management of cardiovascular conditions. In recent years, advances in medical technology, including non-invasive imaging and machine learning (ML) algorithms, have created new opportunities for early detection and diagnosis of CVD [4].

ML algorithms can also be applied to medical imaging data to improve the accuracy and speed of diagnosis. For example, ML models can analyze CT or MRI scans to identify patterns and features associated with CVD. These models can accurately diagnose patients with CVD or determine their current state of health. Additionally, the models provide a probability score for the diagnosis, which can help patients understand their condition better. Machine learning (ML) algorithms, including binary classifiers, have shown promise in improving the accuracy and efficiency of CVD diagnosis. Binary classifiers are ML models that can classify data into two categories, such as the presence or absence of CVD [5]. These algorithms can analyze a diverse range of clinical and imaging data to accurately pinpoint patterns and features that are associated with the onset and progression of CVD. In addition to diagnosis, ML algorithms can also be used for risk prediction and personalized treatment of CVD. For example, ML models can predict the likelihood of CVD development or recurrence and recommend appropriate preventive measures or treatment options based on patient-specific characteristics. The healthcare industry considers data mining essential and utilizes machine learning techniques to create models that classify necessary information [6]. These models can use descriptive functions like clustering to uncover new insights or prediction techniques like classification to anticipate chronic diseases such as cardiovascular issues.

The objective of this study is to evaluate and compare the performance of seven machine learning models for cardiovascular disease (CVD) prediction: Random Forest (RF), Logistic Regression (LR), Naive Bayes (NB), k-Nearest Neighbors (kNN), Support Vector Machines (SVM), Gradient Boosting (GB), and Artificial Neural Networks (ANN). These models are trained and tested using clinical data samples, with their efficiency assessed through metrics such as accuracy, precision, recall, F1-score, and Area Under the Receiver Operating Characteristic Curve (AUC-ROC).

Despite significant advances in CVD prediction, there is limited research systematically comparing machine learning models, examining the effects of hyperparameter tuning, and addressing class imbalance while identifying key personal and clinical attributes. By leveraging advanced techniques, including hyperparameter optimization and class balancing methods, this study aims to bridge these gaps and deliver actionable insights into improving early diagnosis and prevention of CVD.

To establish a foundation for this analysis, the following section reviews prior works that highlight methodologies, datasets, and challenges in CVD prediction using machine learning. This review underscores the current limitations in the field and situates this study within the broader context of ongoing research efforts.

This study systematically evaluates seven machine learning models for predicting cardiovascular disease. By utilizing advanced preprocessing techniques, such as SMOTE–ENN for handling class imbalance and Grid Search Cross-Validation for hyperparameter optimization, the study aims to improve predictive accuracy and robustness. The findings are compared with prior research to highlight advancements and provide actionable insights for clinical applications.

To contextualize this research, the next section reviews key studies on machine learning applications in cardiovascular disease prediction, focusing on methodologies, datasets, and challenges.

### Related Work

Numerous studies have explored the application of machine learning (ML) methods for cardiovascular disease (CVD) prediction, employing diverse datasets and algorithms. These works provide valuable insights but often face challenges related to data quality, class imbalance, and limited algorithm comparisons. The following review highlights key contributions and their limitations, providing context for the present study.

Lupague et al. [7] utilized the BRFSS 2021 dataset to evaluate machine-learning models for CVD prediction. Logistic Regression emerged as the top-performing model, with an AUC of 0.837. However, the study relied on imbalanced data and did not explore advanced ML techniques or hyperparameter optimization, limiting its predictive potential.

Bhatt et al. [8] applied classifiers such as Decision Tree, Random Forest, and Multilayer Perceptron (MLP) on a Kaggle dataset. MLP achieved the highest accuracy (87.28%) after hyperparameter tuning. Despite these results, the study did not address data imbalance, and the dataset lacked diversity, which could limit its generalizability.

Kadhim et al. [9] evaluated algorithms, including Random Forest and Support Vector Machines (SVM), using IEEE DataPort datasets. Random Forest achieved the highest performance (accuracy = 95.4%) after random search optimization. However, the study focused on a single dataset and did not explore cross-validation with alternative datasets.

Sinha et al. [10] tested classifiers such as Logistic Regression, k-Nearest Neighbor (kNN), and Random Forest on the Cleveland Heart Disease dataset. Random Forest outperformed other models, achieving an accuracy of 87%. However, the study did not employ advanced preprocessing techniques, such as data balancing, to address class imbalance.

Akkaya et al. [11] analyzed the BRFSS 2020 dataset using eight ML classifiers. To handle data imbalance, they employed SMOTE–Tomek Links, achieving promising results with XGBoost (accuracy = 89%). While the study addressed imbalance, it lacked a detailed comparison of hyperparameter optimization methods.

Despite significant progress, prior research faces notable limitations, including insufficient handling of class imbalance, limited algorithm comparisons, and a lack of systematic hyperparameter optimization. This study addresses these gaps by evaluating seven machine-learning models on the BRFSS 2021 dataset. Advanced preprocessing techniques, such as SMOTE–ENN and systematic hyperparameter tuning using Grid Search Cross-Validation, are employed to enhance predictive performance and ensure robust evaluation. These methods provide actionable insights for improving CVD prediction and applicability in real-world healthcare settings.

## 2. Materials and Methods

### 2.1. Study Design

This study evaluated the performance of seven machine learning models for cardiovascular disease (CVD) prediction. The analysis followed a systematic workflow, starting with the use of the BRFSS 2021 dataset, which included clinical and behavioral features relevant to CVDs.

Data preprocessing included handling missing values, normalizing features, and addressing class imbalance through SMOTE–ENN. Seven classification models—Random Forest, Logistic Regression, Naive Bayes, k-Nearest Neighbors, Support Vector Machines, Gradient Boosting, and Artificial Neural Networks—were trained and tested.

Model performance was assessed using standard metrics such as accuracy, precision, recall, specificity, F1-score, and AUC-ROC.

**Figure 1:**
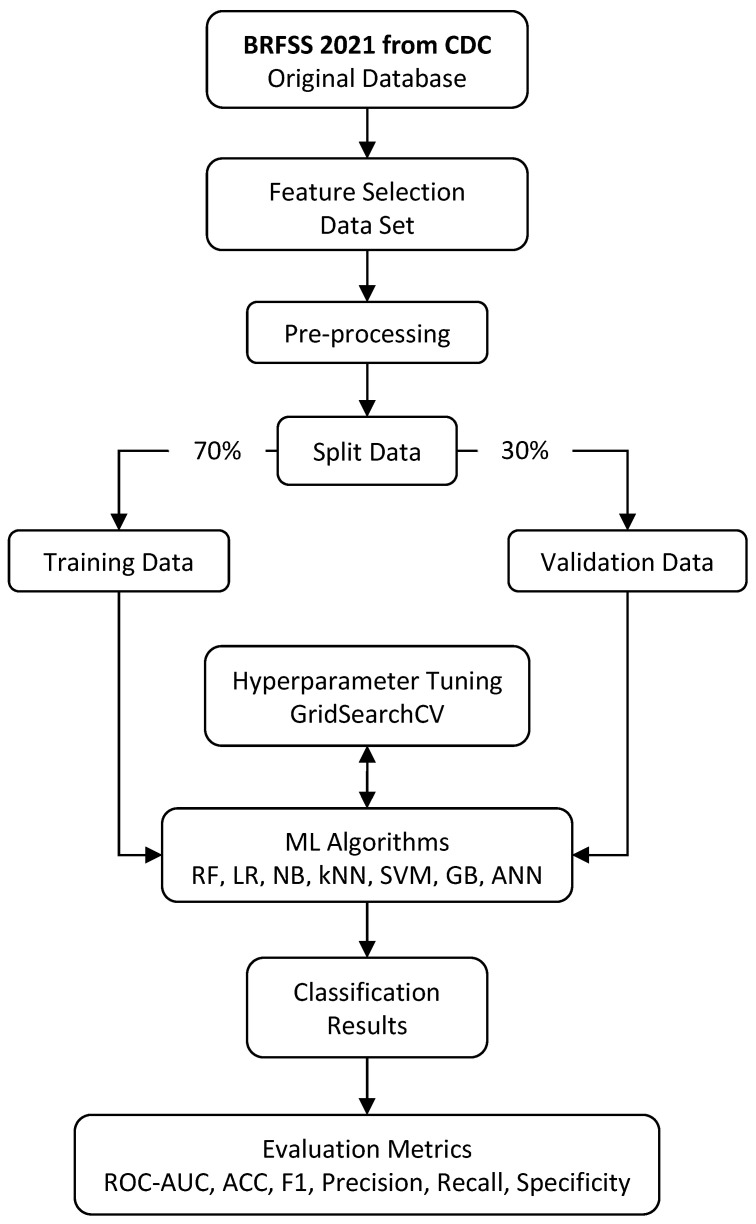
Study architecture workflow illustrating the key steps.

The overall workflow of the study is depicted in Figure 1. This figure outlines the key steps, starting from data acquisition and preprocessing to model training and evaluation. The study was designed to systematically evaluate the predictive performance of multiple machine learning algorithms while addressing challenges such as class imbalance and feature engineering. This structured approach ensures consistency and reproducibility in the analysis process.

### 2.2. Data Collection

The data collection process for this study involved accessing the annual BRFSS 2021 dataset obtained from the Centers for Disease Control and Prevention (CDC) [12]. The dataset originally contained 438,693 records and 304 attributes.

The selection of 18 attributes was guided by their clinical relevance to cardiovascular disease prediction (Table 1). These attributes included key demographic, behavioral, and health-related factors such as age, smoking habits, physical activity levels, and body mass index (BMI), which are known to significantly influence cardiovascular health outcomes. The data were accessed locally.

After filtering the data based on these attributes, the number of records was reduced to 308,854 instances for analysis and model development. The dataset was reduced from its original size of 438,693 records to 308,854 records after excluding instances with missing or inconsistent data. This preprocessing step ensured the integrity and reliability of the dataset for subsequent analysis.

Categorical variables were encoded into numerical representations to ensure compatibility with the requirements of machine learning algorithms.

### 2.3. Pre-Processing

Data preprocessing prepared the raw data for analysis and modeling. This process included data cleaning, handling missing values, addressing outliers, and resolving inconsistencies in the dataset. Whether for supervised or unsupervised learning, the manipulation of attributes or instances was applied to enhance model performance [13].

The initial data cleaning process began with the removal of 80 duplicate rows, ensuring the integrity of the dataset. Outlier treatment was performed based on clinically informed thresholds for key attributes. For example, height values outside the range of 140 cm to 210 cm and weight values below 45 kg or exceeding 200 kg were excluded. These thresholds were established to align with typical human physiological ranges, minimizing the impact of extreme values on model training. As a result, the dataset was refined to include 306,939 rows, ensuring a reliable foundation for subsequent analysis and modeling.

The use of additional attributes in creating the classifier generally resulted in higher accuracy, as evidenced by the correlation found between attribute quantity and accuracy [14]. To enhance the model further, feature engineering was performed by adding the body mass index (BMI), which was calculated from the height (cm) and weight (kg) attributes. This addition provided the classifier with more comprehensive information, potentially improving its predictive performance and robustness.

An investigation of the dataset revealed a significant class imbalance in the target variable, heart disease, with 91.91% of instances belonging to class 0 (absence of heart disease) and 8.09% to class 1 (presence of heart disease Table 2). This imbalance posed challenges in developing predictive models, as it could result in a bias toward the majority class. Addressing this imbalance was necessary to ensure the model’s accuracy and effectiveness in predicting instances of heart disease across both classes [15].

To address the class imbalance, the SMOTE–ENN (Synthetic Minority Oversampling Technique followed by Edited Nearest Neighbors) method was employed. Synthetic samples for the minority class were generated using SMOTE, thereby balancing the dataset. The ENN technique was then applied to refine the dataset by removing noisy examples. By employing SMOTE–ENN, a more representative dataset was created, leading to improved model performance in predicting instances of heart disease across both classes.

Following data cleaning, feature engineering, and addressing class imbalance, the next step was data transformation. This process involved the normalization of features using min–max scaling to ensure similar scales and distributions. Normalization was particularly important for models sensitive to the scale of input variables, such as Support Vector Machines and k-Nearest Neighbors [16]. Min–max scaling transformed the values of features to a range between 0 and 1, as shown in Equation (1).

(1) $$X_{scaled}=\frac{X-X_{min}}{X_{max}-X_{min}}\;$$

By applying these techniques, potential biases resulting from varying scales were addressed, optimizing the performance of the models across different algorithms.

A visualization of the distribution of feature attributes was conducted using heatmaps, which provided an intuitive and visually effective method to illustrate the relationships between variables by showcasing their correlation matrix. The body mass index (BMI), derived from the height (cm) and weight (kg) attributes, was identified as a highly significant feature. Additionally, factors such as diabetes, checkups, arthritis, and weight (kg) were found to be the most influential in predicting the likelihood of cardiovascular disease (CVD).

Finally, after completing all preprocessing steps and exploratory data analysis, the dataset was divided into training and testing subsets. This division was implemented with a ratio of 70:30, ensuring that 70% of the data was allocated for training the models while the remaining 30% was reserved for testing their performance.

### 2.4. Implementation and Optimization of Classification Models

The primary goal of a classification algorithm was to identify the correlation between the input variables and the target variable. To make predictions, seven supervised machine learning (ML) models were employed. The performance of these algorithms depended on factors such as feature selection, data quality, class imbalance, hyperparameter tuning, and overfitting [17].

Random Forests (RF)—Leveraging Grid Search Cross-Validation (GridSearchCV), the model’s hyperparameters were optimized, identifying the optimal configuration: ‘max_depth’ = None, ‘min_samples_leaf’ = 1, ‘min_samples_split’ = 2, and ‘n_estimators’ = 300. This optimization process involved exhaustively exploring a specified grid and evaluating the model’s performance through cross-validation. By incorporating these optimal parameters, the effectiveness of the model was improved, enhancing its capacity to generalize and make precise predictions on new data.

Logistic Regression (LR)—The model was optimized using Grid Search Cross-Validation (GridSearchCV), identifying the optimal configuration: solver = ‘saga’, penalty = ‘l1’, max_iter = 500, class_weight = none, and C = 0.08858667904100823. This optimization process involved an exhaustive search for optimal hyperparameters, with model performance evaluated through cross-validation. Incorporating these optimal parameters improved the model’s performance and enhanced its generalization capability on unseen data.

Naive Bayes (NB)—The parameters var_smoothing = 1 × 10^−7^ and priors = [0.3, 0.7] for the Gaussian Naive Bayes model were obtained using Grid Search Cross-Validation (GridSearchCV). This method enabled hyperparameter optimization by exploring a predefined grid of values and evaluating model performance through cross-validation techniques.

K-Nearest Neighbors (kNN)—The model was configured with the parameters: metric = ‘Euclidean’, n_neighbors = 2, and weights = ‘uniform’, which were obtained through the fine-tuning process using Grid Search Cross-Validation (GridSearchCV). The metric parameter specified the distance metric used to calculate the distance between data points, with ‘Euclidean’ representing the Euclidean distance. With n_neighbors set to 2, the model considered the two nearest neighbors when making predictions. Additionally, the weights parameter was set to ‘uniform’, indicating that all neighbors contributed equally to the classification decision. This configuration enabled the kNN classifier to effectively classify data points based on their proximity to other points in the feature space.

Support Vector Classifier (SVC)—The model was configured with the parameters: kernel = ‘rbf’, random_state = 42, and probability = True. The ‘rbf’ kernel was chosen to transform the input data into higher-dimensional space, enabling the model to capture complex relationships within the data. The random_state parameter was set to 42 to ensure reproducibility by fixing the random seed. Additionally, setting probability to True allowed the model to output class probabilities, facilitating nuanced decision-making processes. These configurations enabled the SVC model to effectively classify data, make informed predictions, maintain reproducibility, and provide probability estimates.

Gradient Boosting (XGB)—The XGBoost classifier was fine-tuned with the best parameters derived from an optimization process using Grid Search Cross-Validation (GridSearchCV). The model was configured with the following parameters: colsample_bytree = 1.0, learning_rate = 0.2, max_depth = 7, n_estimators = 300, and subsample = 0.9, achieving a balance between complexity and generalization. Regularization was enforced through reg_alpha = 0.5 and reg_lambda = 0.5 to mitigate overfitting. By integrating these parameters, the XGBoost classifier effectively handled complex datasets, delivering robust predictions while maintaining interpretability and generalization capability.

Artificial Neural Network (ANN)—The model employed was the Keras Sequential, designed to tackle binary classification tasks. Configured to prevent overfitting while optimizing performance, this architecture incorporated multiple layers to capture and extract significant features from the input data. The initial layer consisted of a dense layer with 128 units, activated by the ReLU function, followed by a dropout layer with a rate of 0.3 to mitigate overfitting. Batch normalization was applied to normalize the activations. Subsequently, another dense layer with 64 units and ReLU activation was included, followed by another dropout layer and batch normalization. Finally, a single-unit dense layer with sigmoid activation was incorporated to produce binary classification output.

The model was compiled with the Adam optimizer and mean squared error (MSE) loss function for optimization. During training, early stopping was implemented to prevent overfitting by halting the process if the validation loss failed to decrease for a specified number of epochs. Through these architectural choices and training procedures, the model achieved accurate classification while minimizing overfitting on the validation set.

### 2.5. Experimental Setup

The simulations were conducted using virtual machines configured to handle the computational demands of the study. This infrastructure provided sufficient resources to execute the experiments efficiently and reliably, ensuring accurate and reproducible results.

### 2.6. System Architecture

**Figure 2:**
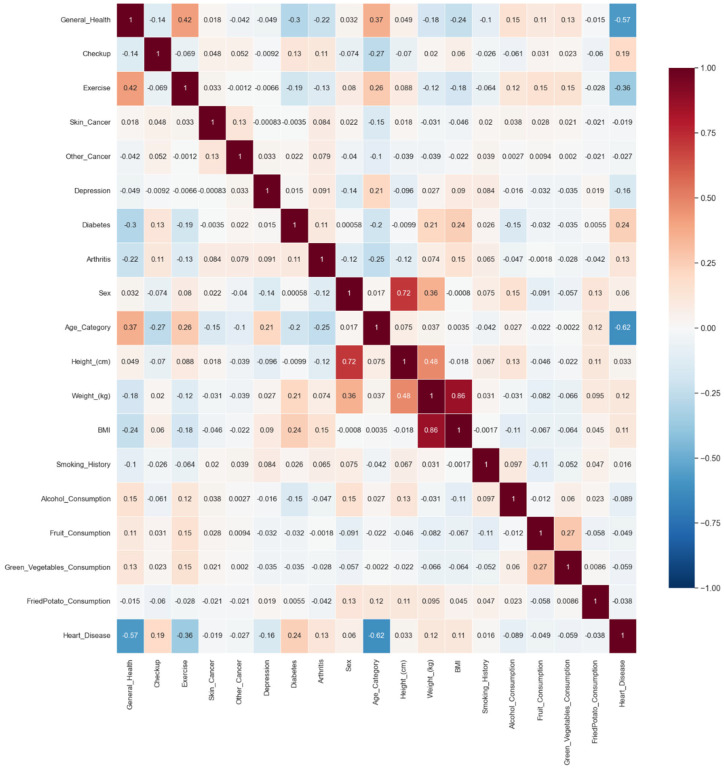
Correlation heatmap of selected features.

In this study, the system architecture followed a structured workflow (Figure 2), beginning with data acquisition and feature selection. After the data was obtained, preprocessing steps were applied to clean and organize it for effective analysis. The dataset was then divided into training and validation sets with a 70:30 ratio. Various machine learning algorithms, including Random Forests, Logistic Regression, Naive Bayes, K-Nearest Neighbors, Support Vector Machines, Gradient Boosting, and Artificial Neural Networks, were trained and assessed within this framework.

Hyperparameter tuning was an important component, achieved through Grid Search Cross-Validation (GridSearchCV) [18]. This technique systematically explored a predefined parameter grid to identify the optimal configuration for each algorithm. By leveraging GridSearchCV, the study ensured that each model was fine-tuned for maximum predictive accuracy and generalization capability. Once trained, the models were evaluated for their accuracy in predicting target outcomes.

In this study, preprocessing, hyperparameter tuning, and model evaluation were used to successfully assess the predictive efficacy and generalization capacity of each method. The process involved fine-tuning the preprocessing steps to optimize data quality and relevance, adjusting hyperparameters to enhance model performance, and subjecting the models to rigorous evaluation metrics to gauge their effectiveness in real-world scenarios.

The model development and testing process was implemented in the Spyder IDE, with Python as the primary programming language. Key libraries such as Pandas for data handling, NumPy for numerical computations, TensorFlow for deep learning, and Scikit-Learn for machine learning were incorporated to streamline data processing, model training, and evaluation.

### 2.7. Evaluation Metrics

To assess prediction models, six widely used evaluation measures were employed. These included precision, recall, specificity, accuracy (ACC), F1-score, and Area Under the ROC Curve (ROC-AUC). These metrics are frequently used in various applications, such as disease diagnosis, fraud detection, and spam filtering, to evaluate the performance of binary classifiers.

(2) $$Precision=\frac{TP}{TP+FP}\;$$

(3) $$Recall=\frac{TP}{TP+FN}\;$$

(4) $$Specificity=\frac{TN}{TN+FP}\;$$

(5) $$Accuracy=\frac{TN+TN}{TP+TN+FP+FN}\;$$

(6) $$F1-score=2\times \frac{Precision\times Recall}{Precision+Recall}\;$$

(7) $$ROC-AUC=\int_{0}^{1}TPR(FPR)d(FPR)\;$$

where:

- *TP* is true positive.
- *TN* is true negative.
- *FP* is false positive.
- *FN* is false negative.
- *TPR* = $\frac{TP}{TP+FN}$ is true positive rate.
- *FPR* = $\frac{FP}{FP+TN}$ is false positive rate.

### 2.8. Software

Spyder IDE version 6.0.1 with Python version 3.12.7 served as the primary platform for creating the programs and conducting hyperparameter tuning to optimize the performance of the classifiers. This IDE provided a user-friendly interface for coding, debugging, and executing Python scripts efficiently. By leveraging Spyder’s capabilities, various machine learning algorithms were implemented, their hyperparameters fine-tuned using techniques such as Grid Search Cross-Validation (GridSearchCV), and the models’ performance was thoroughly evaluated.

## 3. Results

After training, the models’ performance was assessed using the validation dataset, enabling a direct comparison of their predictive capabilities. This evaluation provided important insights into the effectiveness of each model and its ability to generalize to unseen data.

Table 3 summarizes the performance of all classification models across various metrics, including AUC, accuracy, F1-score, precision, recall, and specificity. This format provided a comprehensive overview of the models’ strengths and weaknesses, enabling a clear comparison of their predictive capabilities.

The table presented the weighted average scores for each method, including metrics such as AUC (Area Under the Curve), accuracy (ACC), F1-score, precision, recall, and specificity. The scores showed that each method performed differently across various metrics. For instance, the k-Nearest Neighbors (kNN) method demonstrated the strongest performance in classifying the data, scoring the highest across all metrics. Conversely, the Naive Bayes method exhibited the lowest scores, indicating relatively weaker performance.

**Figure 3:**
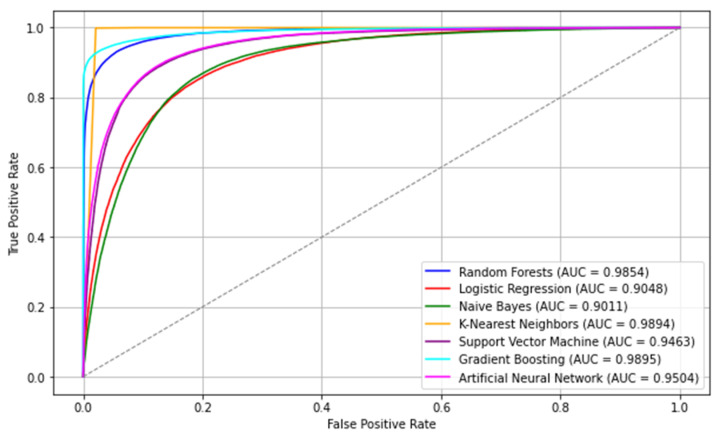
Receiver Operating Characteristic (ROC) Curve illustrating the discriminatory power (AUC) of classification models.

Figure 3 displays the Receiver Operating Characteristic (ROC) Curve outcomes of the machine learning models. From this figure, it was observed that the Random Forest, k-Nearest Neighbors (kNN), and Gradient Boosting models exhibited superior performance, as evidenced by their higher AUC-ROC scores compared to other models. These results suggested that these models had strong discriminatory power and were effective in accurately classifying the data.

Each model in the evaluation exhibited varying degrees of performance across different metrics, highlighting their respective strengths and weaknesses. The kNN model stood out with the highest accuracy and F1-score, indicating its effectiveness in making accurate predictions across both positive and negative classes. In comparison to other machine learning algorithms, Random Forests and Gradient Boosting displayed excellent performance in distinguishing between positive and negative instances, as evidenced by their high AUC scores. Overall, their superior performance in terms of AUC highlighted their potential as powerful tools for data analysis and decision-making across various fields.

Logistic Regression, Naive Bayes, and Artificial Neural Network showed moderate performance across multiple metrics, suggesting a balanced yet improvable classification accuracy. In contrast, SVM lagged behind with relatively lower scores across most metrics, indicating its limitations in this specific classification task. This diverse range of performance emphasized the critical need to select a model precisely tailored to the objectives and requirements of the classification problem, underscoring the importance of choosing the right model to align with the specific goals and needs of the task.

**Figure 4:**
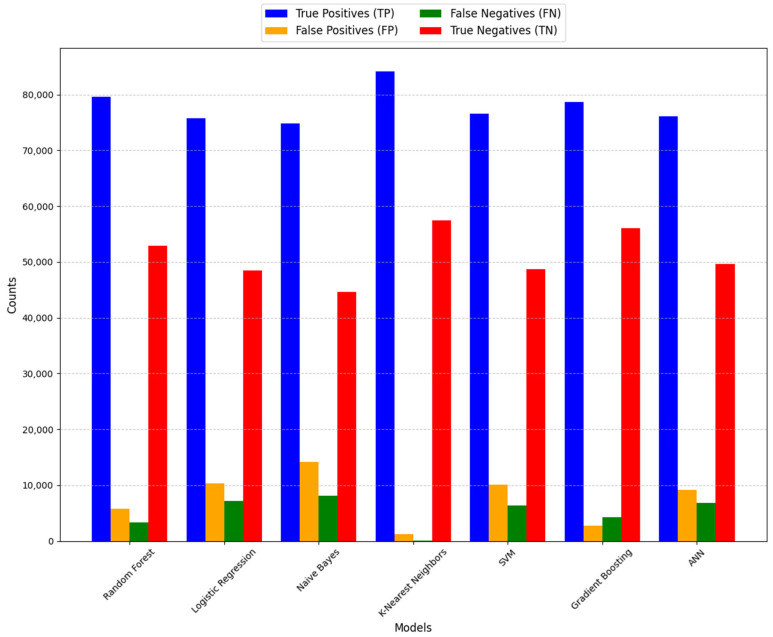
Confusion matrices of classification models, showing true positives, true negatives, false positives, and false negatives for each model.

Figure 4 presents the confusion matrix metrics, including true positives (TP), false positives (FP), false negatives (FN), and true negatives (TN), for all classification models.

Starting with models that exhibited relatively weaker performance, Logistic Regression, Naive Bayes, and Artificial Neural Networks demonstrated moderate classification accuracy. These models showed comparable true positive (TP) and true negative (TN) rates but had slightly higher false positive (FP) and false negative (FN) counts. This indicated a decent ability to classify instances, though with a tendency to misclassify a notable number of instances.

In contrast, the k-Nearest Neighbors (kNN) model showcased significantly lower FP and FN values, highlighting its robustness in minimizing misclassifications. Support Vector Machine (SVM) exhibited a performance pattern similar to Logistic Regression and Naive Bayes, with balanced TP and TN rates but slightly higher FP and FN counts. However, Random Forest and Gradient Boosting models stood out with consistently balanced TP and TN counts, suggesting superior classification accuracy and effectiveness in handling both positive and negative instances.

Among the models considered, the kNN model emerged as particularly effective due to its capacity to reduce misclassifications and attain a well-balanced classification accuracy. This effectiveness was attributed to its inherent mechanism of leveraging the proximity of data points, enabling it to make accurate predictions by considering the similarities between instances in the dataset. Therefore, the kNN model emerged as a promising choice for classification tasks, as minimizing errors and ensuring balanced classification performance were critical objectives.

Overall, this analysis laid the foundation for subsequent discussions and conclusions, guiding future research directions and applications in the field of machine learning and data analysis.

## 4. Discussion

In this paper, we have evaluated seven classification models: Random Forest, Logistic Regression, Naive Bayes, k-Nearest Neighbors (kNN), Support Vector Machine (SVM), Gradient Boosting, and Artificial Neural Network (ANN). Each model was assessed based on various performance metrics such as accuracy, Area Under the Curve (AUC), F1-score, precision, recall, and specificity. These evaluations were complemented by advanced preprocessing and optimization techniques, which enhanced the reliability and robustness of the results.

The results of this study demonstrate the predictive power of machine learning algorithms in identifying cardiovascular disease risks. Starting with the weighted average scores, which provide an overview of the overall performance of each method across multiple metrics, we can observe that kNN achieved the highest scores across all metrics. This highlights the effectiveness of advanced preprocessing techniques, such as SMOTE–ENN, and the impact of fine-tuning hyperparameters in enhancing model performance. Conversely, Naive Bayes exhibited lower scores across the board, indicating comparatively weaker performance across all evaluated metrics, particularly in handling complex data distributions.

In this study, several methodological improvements contributed to the superior performance of k-Nearest Neighbor (kNN) compared to previous works (Table 4). First, we utilized SMOTE–ENN, an advanced technique for handling class imbalance, which effectively reduced noise in the dataset and improved classification robustness. Second, hyperparameter optimization through Grid Search Cross-Validation enabled the fine-tuning of key parameters, such as the number of neighbors (k) and the distance metric, ensuring optimal model configuration. While these advancements significantly enhanced kNN’s accuracy and reliability in predicting cardiovascular disease, the potential introduction of synthetic noise through SMOTE–ENN and the dependency on predefined parameter grids remain challenges to be addressed in future studies.

When examining the ROC curve, outcomes displayed in Figure 4, we observe that Random Forest, kNN, and Gradient Boosting models exhibit superior performance, as evidenced by their higher AUC-ROC scores compared to other models. These results highlight the strong discriminatory power of these algorithms, making them particularly effective in distinguishing between individuals at risk of cardiovascular disease and healthy individuals. Such performance is very important in clinical settings where accurate risk stratification can significantly impact early intervention and treatment planning.

**Figure 5:**
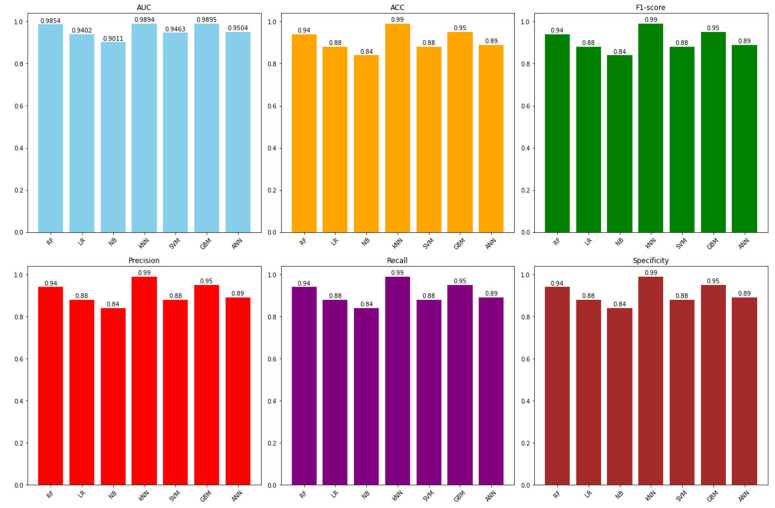
Classification Model Performance Based on Metrics.

Furthermore, the comparison of classification model performance presented in Figure 5 highlights the relative strengths of each algorithm across various metrics.

kNN stands out with the highest accuracy and F1-score, indicating its effectiveness in making accurate predictions across both positive and negative classes. Random Forest and Gradient Boosting, on the other hand, demonstrate superior performance in terms of AUC, reflecting their proficiency in distinguishing between positive and negative instances. However, the interpretability of these advanced models remains a challenge, emphasizing the need for transparent approaches when integrating machine learning tools into clinical decision-making.

Analyzing the confusion matrix of the seven machine learning algorithms reveals interesting insights into the performance of each model. For instance, Logistic Regression, Naive Bayes, and Artificial Neural Network demonstrate moderate classification accuracy but tend to misclassify a notable number of instances, as indicated by their higher false positive and false negative counts. In contrast, kNN showcases significantly lower false positive and false negative values, emphasizing its robustness in minimizing misclassifications. This capability is particularly relevant in healthcare applications, where reducing classification errors can directly impact patient outcomes by improving diagnostic accuracy and enabling timely interventions.

Overall, the results suggest that each classification model has strengths and weaknesses, and the choice of the most appropriate model depends on the specific requirements and objectives of the classification task. The insights gained from this study highlight the importance of aligning model selection with clinical needs and balancing accuracy, interpretability, and computational efficiency. Future research should explore the integration of these models into real-world healthcare workflows and assess their practical impact and scalability in diverse clinical settings.

Table 4 summarizes a comparative analysis of previous research studies, their proposed models, and their reported performance metrics. This comparison provides valuable context for evaluating the effectiveness of the models tested in this study.

Among the models tested in our study using the BRFSS 2021 dataset, the k-Nearest Neighbors model demonstrated the highest accuracy, achieving a remarkable score of 99.06%. This performance surpasses the results reported in previous studies, where accuracies ranged between 79.18% and 91.57%, as seen in Table 4. The exceptional performance of the k-Nearest Neighbors model highlights the impact of advanced preprocessing techniques, such as SMOTE–ENN and hyperparameter tuning, which were rigorously applied in this study.

These results emphasize the potential of k-Nearest Neighbors for predictive modeling in cardiovascular disease, offering significant improvements over models proposed in prior research. Furthermore, the methodology employed in this study demonstrates the importance of balancing data preprocessing and model optimization in achieving high accuracy and reliability in healthcare applications.

## 5. Conclusions

This study demonstrates the significant potential of machine learning models in predicting cardiovascular disease (CVD) with high accuracy. By evaluating multiple binary classification algorithms—Random Forests, Logistic Regression, Naive Bayes, k-Nearest Neighbors (kNN), Support Vector Machines (SVM), Gradient Boosting, and Artificial Neural Networks (ANN)—the kNN model emerged as the most accurate and reliable method for CVD prediction.

The kNN model achieved an accuracy rate of 99.06%, surpassing previous studies and establishing a new benchmark in CVD prediction. In addition to this exceptional accuracy, the kNN model demonstrated strong performance across other evaluation metrics, including F1-score, precision, recall, and ROC-AUC, underscoring its robustness and reliability in identifying individuals at risk of CVD.

Comparative analysis with prior studies highlights the advancements achieved in this research. For example, Mamun M. et al. and Akkaya B. et al. reported accuracies of 91.57% and 89.00%, respectively, using different machine-learning models [18,19]. In contrast, the kNN model in our study achieved an accuracy of 99.06%, reflecting the effectiveness of our preprocessing techniques, feature engineering, and hyperparameter tuning.

The high accuracy achieved by the kNN model can be attributed to key methodological enhancements, including the handling of class imbalance through the SMOTE–ENN technique and hyperparameter optimization using Grid Search Cross-Validation. These steps ensured that the models were both accurate and robust, enhancing their applicability to real-world scenarios.

The findings of this study have significant implications for clinical practice. Integrating the kNN model into healthcare systems could enhance the early detection and diagnosis of CVD, ultimately improving patient outcomes. Accurate predictive models, like the kNN model, can play a critical role in reducing complications and mortality associated with cardiovascular diseases through timely interventions.

Future research should aim to integrate additional features and explore advanced methods, such as deep learning, to further improve predictive accuracy. Enhancing the interpretability of these models will also be essential for supporting clinicians in understanding the factors driving predictions, thereby increasing their trust and usability in healthcare settings. Moreover, leveraging predictors such as job satisfaction and emotional exhaustion may provide further insights into health risks, as previous studies have identified links between burnout, personality traits, and disease occurrence [20,21].

This study establishes the kNN model as a highly accurate and reliable method for predicting cardiovascular diseases, achieving a groundbreaking accuracy rate of 99.06%. This result sets a new benchmark in CVD prediction, paving the way for more effective and efficient early detection strategies that have the potential to save lives and enhance patient care.

## Figures and Tables

**Table 1:** Data set description.

Field	Values
General_Health	Categorical Excellent—4, Very Good—3, Good—2, Fair—1, Poor—0
Checkup	Categorical Within the past year—4, Within the past 2 years—3, Within the past 5 years—2, 5 or more years ago—1, Never—0
Exercise	Categorical Yes—1, No—0
Skin_Cancer	Categorical Yes—1, No—0
Other_Cancer	Categorical Yes—1, No—0
Depression	Categorical Yes—1, No—0
Diabetes	Categorical Yes—2, Yes, but female told only during pregnancy—2, No, pre-diabetes or borderline diabetes—1, No—0
Arthritis	Categorical Yes—1, No—0
Sex	Categorical Male—1, Female—0
Age_Category	categorical 18:24—12, 25:29—11, 30:34—10, 35:39—9, 40:44—8, 45:49—7, 50:54—6, 55:59—5, 60:64—4, 65:69—3, 70:74—2, 75:79—1, 80+—0
Height_(cm)	Numerical
Weight_(kg)	Numerical
Smoking_History	Categorical Yes—1, No—0
Alcohol_Consumption	Numerical
Fruit_Consumption	Numerical
Green_Vegetables_Consumption	Numerical
FriedPotato_Consumption	Numerical
Heart_Disease	Categorical Yes—1, No—0

**Table 2:** Distribution of heart disease cases in the dataset.

	Class 0	Class 1	Rows
Before SMOT–EEN	282,136	24,803	306,939
After SMOT–EEN	195,926	276,559	472,485

**Table 3:** Weighted average scores of classification models across performance metrics.

Method	AUC	ACC	F1	Precision	Recall	Specificity
Random Forest	0.9854	0.94	0.94	0.94	0.94	0.94
Logistic Regression	0.9402	0.88	0.88	0.88	0.88	0.88
Naive Bayes	0.9011	0.84	0.84	0.84	0.84	0.84
kNN	0.9894	0.99	0.99	0.99	0.99	0.99
SVM	0.9463	0.88	0.88	0.88	0.88	0.88
Gradient Boosting	0.9895	0.95	0.95	0.95	0.95	0.95
Artificial Neural Network	0.9504	0.89	0.89	0.89	0.89	0.89

**Table 4:** Performance of previous work.

Authors (Year)	Dataset Collection (Samples)	Applied Models	Performance (Proposed Model)
Lupague R.M.J.M et al. (2023) [7]	BRFSS 2021 dataset (308,854)	Logistic Regression (proposed), K-Nearest Neighbor, Naive Bayes, Decision Tree Classifier, and Random Forest	Accuracy: 79.18%
Akkaya B. et al. (2022) [11]	BRFSS 2020 dataset (319,795)	Logistic Regression, Support Vector Machines, XGBoost (proposed), Naive Bayes, k-Nearest Neighbor, Decision Tree, Adaboost, and Multilayer Perceptron,	Accuracy: 89.00%
Bhatt C.M. et al. (2023) [8]	Kaggle CVD dataset (70,000)	Decision Tree, Random Forest, Multilayer Perceptron (proposed), and XGBoost	Accuracy: 87.28%
Mamun M. et al. (2022) [19]	BRFSS 2020 dataset (319,795)	XGBoost, AdaBoost, Random Forest, Decision Tree, Naive Bayes, and Logistic Regression (proposed)	Accuracy: 91.57%
Our (2024)	BRFSS 2021 dataset (308,854)	Random Forest, Logistic Regression, Naive Bayes, k-Nearest Neighbor (proposed), Support Vector Machine, Gradient Boosting, and Artificial Neural Network	Accuracy: 99.06%

## Data Availability

The manuscript has not been published elsewhere. Data are contained within the article.

## Abbreviations

ACC	Accuracy
ANN	Artificial Neural Networks
AUC	Area Under the Curve
BMI	Body Mass Index
BRFSS	Behavioral Risk Factor Surveillance System
CDC	Centers for Disease Control and Prevention
CT	Computed Tomography
CVD	Cardiovascular Disease
DT	Decision Tree
ENN	Edited Nearest Neighbors
FN	False Negative
FP	False Positive
FPR	False Positive Rate
GB	Gradient Boosting
GridSearchCV	Grid Search Cross-Validation
kNN	K-Nearest Neighbors
LR	Logistic Regression
ML	Machine Learning
MLP	Multilayer Perceptron
MPI	Myocardial Perfusion Imaging
MRI	Magnetic Resonance Imaging
MSE	Mean Squared Error
NB	Naive Bayes
ReLU	Rectified Linear Unit
RF	Random Forests
ROC	Receiver Operating Characteristic
ROC-AUC	Area Under the Receiver Operating Characteristic Curve
SMOTE	Synthetic Minority Oversampling Technique
SMOTE–ENN	Synthetic Minority Oversampling Technique Edited Nearest Neighbors
SMOTE–Tomek Link	Synthetic Minority Oversampling Technique Tomek Links
SVC	Support Vector Classifier
SVM	Support Vector Machines
SVM	Support Vector Machine
TN	True Negative
TP	True Positive
TPR	True Positive Rate
WHO	World Health Organization
XGB	XGBoost

## References

[1] Cardiovascular Diseases. World Health Organization. https://www.who.int/health-topics/cardiovascular-diseases.

[2] Frąk W., Wojtasińska A., Lisińska W., Młynarska E., Franczyk B., Rysz J. (2022). Pathophysiology of Cardiovascular Diseases: New Insights into Molecular Mechanisms of Atherosclerosis, Arterial Hypertension, and Coronary Artery Disease. Biomedicines.

[3] Sirajuddin A., Chen J.S., White S.C., Truong R.M., Collins J.P., Slomka P.J. (2021). Ischemic heart disease: Noninvasive Imaging Techniques and Findings. RadioGraphics.

[4] Patidar S., Kumar D., Rukwal D., Singari R.M., Kankar P.K. (2022). Comparative Analysis of Machine Learning Algorithms for Heart Disease Prediction. Advances in Transdisciplinary Engineering.

[5] Ananey-Obiri D., Sarku E. (2020). Predicting the Presence of Heart Diseases using Comparative Data Mining and Machine Learning Algorithms. Int. J. Comput. Appl..

[6] Tougui I., Jilbab A., El Mhamdi J. (2020). Heart disease classification using data mining tools and machine learning techniques. Health Technol..

[7] Lupague R.M.J.M., Mabborang R.C., Bansil A.G., Lupague M.M. (2023). Integrated Machine Learning Model for Comprehensive Heart Disease Risk Assessment Based on Multi-Dimensional Health Factors. Eur. J. Comput. Sci. Inf. Technol..

[8] Bhatt C.M., Patel P., Ghetia T., Mazzeo P.L. (2023). Effective Heart Disease Prediction Using Machine Learning Techniques. Algorithms.

[9] Kadhim M.A., Radhi A.M. (2023). Heart disease classification using optimized Machine learning algorithms. Iraqi J. Comput. Sci. Math..

[10] Sinha A., Narula D., Pandey S.K., Pati S., Kumar R. (2024). CARDPSoML: Comparative approach to analyze and predict cardiovascular disease based on medical report data and feature fusion approach. Health Sci. Rep..

[11] Akkaya B., Sener E., Gursu C. A Comparative Study of Heart Disease Prediction Using Machine Learning Techniques. Proceedings of the International Congress on Human-Computer Interaction, Optimization and Robotic Applications (HORA).

[12] BRFSS Survey Data and Documentation. https://www.cdc.gov/brfss/annual_data/annual_2021.html.

[13] Jothikumar R., Sivabalan R.V., Kumarasen A.S. (2015). Data cleaning using weka for effective data mining in health care industries. Int. J. Appl. Eng. Res..

[14] Taha M.A., Alsaidi S.A.A.A., Hussein R.A. Machine Learning Techniques for Predicting Heart Diseases. Proceedings of the the 2022 International Symposium on iNnovative Informatics of Biskra, ISNIB 2022.

[15] Kumar V., Lalotra G.S., Sasikala P., Rajput D.S., Kaluri R., Lakshmanna K., Shorfuzzaman M., Alsufyani A., Uddin M. (2022). Addressing Binary Classification over Class Imbalanced Clinical Datasets Using Computationally Intelligent Techniques. Healthcare.

[16] Galli S. (2023). Feature Scaling in Machine Learning: Standardization, Min-Max Scaling and More. https://www.blog.trainindata.com/feature-scaling-in-machine-learning.

[17] Anderson R., Patel A., Smith C. (2022). Impact of feature selection on machine learning-based cardiovascular disease prediction models. Comput. Biol. Med..

[18] Ahmad G.N., Fatima H., Ullah S., Saidi A.S. (2022). Efficient Medical Diagnosis of Human Heart Diseases Using Machine Learning Techniques with and Without GridSearchCV. IEEE Access.

[19] Mamun M., Uddin M.M., Tiwari V.K., Islam A.M., Ferdous A.U. MLHeartDis: Can Machine Learning Techniques Enable to Predict Heart Diseases?. Proceedings of the 2022 IEEE 13th Annual Ubiquitous Computing, Electronics & Mobile Communication Conference (UEMCON).

[20] Moscu C.-A., Marina V., Dragomir L., Anghele A.-D., Anghele M. (2022). The Impact of Burnout Syndrome on Job Satisfaction among Emergency Department Nurses of Emergency Clinical County Hospital “Sfântul Apostol Andrei” of Galati, Romania. Medicina.

[21] Moscu C.A., Marina V., Anghele M., Anghele A.D., Dragomir L. (2022). Did Personality Type Influence Burn Out Syndrome Manifestations During COVID-19 Pandemic?. Int. J. Gen. Med..

